# Layer and framework theories of lightness

**DOI:** 10.3758/s13414-019-01736-1

**Published:** 2019-05-01

**Authors:** Alessandro Soranzo, Alan Gilchrist

**Affiliations:** 1 Faculty of Social Sciences and Humanities, Sheffield Hallam University, Sheffield, S10 2BP UK; 2 Centre for Behavioural Science and Applied Psychology, Sheffield Hallam University, Sheffield, S1 1WB UK; 3 Department of Psychology, Rutgers University, Newark, NJ 07102 USA

**Keywords:** Lightness, Decomposition, Anchoring, Scaling, Transparency, Layers, Frameworks, Gamut compression, Articulation

## Abstract

Lightness (the perceived dimension running from black to white) represents a problem for vision science because the light coming to the eye from an object totally fails to specify the shade of gray of the object, due to the confounding of surface gray and illumination intensity. The two leading approaches, decomposition theories and anchoring theories, split the retinal image into overlapping layers and adjacent frameworks, respectively. Because each approach has important strengths and some weaknesses, an integration of them would mark an important step forward for the lightness theory. But the problem remains how this integration can actually be realized.

## Introduction: The genesis of the lightness debate

Lightness is the perceived dimension running from black to white. Surface lightness is a basic property of an object, along with size and motion. Yet an understanding of how lightness is computed by the brain is far from complete. The problem is that because the light coming to the eye from an object depends on both the amount of light the surface reflects (reflectance) and the intensity of the illumination, it totally fails to specify the shade of gray of the object. Nevertheless, surface lightness tends to remain constant even when the illumination changes; this is referred to as lightness constancy.

Modern theories of lightness constancy can be dated to the late nineteenth century, when a controversy broke out between, on the one hand, Hering (1878), who supported a low-level explanation of lightness phenomena based on processes sensitive to contrast and, on the other hand, von Helmholtz (1925), who favored a high-level explanation based on processes involving unconscious inference. Helmholtz argued that, in addition to sensing the luminance of a target surface, the visual system unconsciously estimates the illumination level and it is the relationship between target luminance and estimated illumination that predicts the lightness percept. Hering argued that Helmholtz’s appeal to cognition is unnecessary and that low-level processes like adaptation, pupil size, and lateral inhibition can explain the approximate lightness constancy that exists.

David Katz (1935) was the first to study lightness constancy and its failures systematically. In his most widely emulated method, subjects were asked to adjust the gray level of a disk in bright illumination to make it appear equal in lightness to another disk in shadow. He found both constancy and systematic deviations from constancy. The matched level of the two disks was closer to a reflectance match (100% constancy) than to a luminance match (0% constancy), although the gray disk in shadow appeared darker than the disk in brighter illumination.

During the 1920s and 1930s the Gestalt Theorists turned their attention to lightness perception, making a series of important contributions. They emphasized the importance of relative luminance, anticipating Wallach’s (1948) later finding that two disks of different luminance appear equal in lightness when they have the same disk/background luminance ratio. They rejected both Hering’s low-level account and Helmholtz’s high-level theory, arguing that Hering and Helmholtz shared the same two-stage approach featuring a primary stage of raw sensations followed by a second stage in which those sensations were interpreted based on experience, and suggesting that such a theory is not falsifiable.

According to Gelb (1929, excerpted in Ellis, 1938, p. 206): “The essentially problematic aspect of the phenomenon has invariably been taken to be the discrepancy between the ‘stimulus’ and ‘colour’ reaction. Assuming that retinal stimuli and colour-vision stood in a more or less direct correspondence with one another, any departure from this primitive and self-evident relationship – i.e. any ‘discrepancy’ – was explained on empiristic grounds. Thus, if the discrepancy would not be rendered comprehensible by reference to ‘physiological’ (peripheral) factors alone, ‘psychological’ factors would also be invoked. In this way the phenomena of colour constancy were classified as the product of central processes operating upon and reorganizing genetically simpler colour-processes."

The Gestaltists put forward the first mid-level theory. Köhler (1947, p. 103) wrote, “Our view will be that, instead of reacting to local stimuli by local and mutually independent events, the organism responds to the pattern of stimuli to which it is exposed; and that this answer is a unitary process, a functional whole which gives, in experience, a sensory scene rather than a mosaic of local sensations. Only from this point of view can we explain the fact that, with a constant local stimulus, local experience is found to vary when the surrounding stimulation is changed.”

The Gestaltists were well ahead of their time, anticipating concepts that would come to be seen as important only after the computer revolution. Katona (1929), Gelb (1929), Wolff (1933), and Kardos (1934) demonstrated the critical role of depth perception in lightness. The later enthusiasm for explanations based on lateral inhibition at the retina would be possible only by neglecting this work (Soranzo, 2015).

Koffka (1935) and Kardos (1934) proposed that fields of illumination are treated as frames of reference for computing lightness, providing more concreteness to Helmholtz’s notion of estimating illumination level. Koffka (1935, p. 245) emphasized the crucial role of relative luminance at edges (gradients), writing “Our theory of whiteness constancy, will be based on this characteristic of colours, which we found confirmed in so many passages, that perceived qualities depend upon stimulus gradients.” And Koffka made a distinction between luminance gradients that represent reflectance borders and that represent illumination borders. Koffka also spoke of a complementary relationship between lightness and perceived illumination.

### Gestalt theory abandoned

At the end of World War II, the center of scientific work shifted from Europe to the USA, and the Gestalt contributions were pushed aside. A low-level explanation was particularly in vogue during the 1960s mainly because of the physiological discovery of the lateral inhibition process in the limulus (horseshoe crab) retina (Hartline, Wagner, & Ratliff, 1956). Later, however, during the so-called cognitive revolution, the low-level approach was challenged by a series of newfound visual phenomena directly contradicting a retinal interaction explanation. Experiments showed that a change in perceived depth could shift the lightness of a target surface virtually from black to white, with no change in the retinal image (Adelson, 1993; Gilchrist, 1977, 1980), or that a dramatic change in the lightness of a target could be produced merely by causing a (spatially remote) reflectance boundary to appear as an illumination boundary (Gilchrist et al., 1983).

The discovery of these visual phenomena led to the emergence of several inverse-optics, or decomposition, theories (Adelson, 1993; Adelson and Pentland, 1996; Arend, 1994; Bergström, 1977; Barrow & Tenenbaum, 1978; Gilchrist, 1977, 1979; Gilchrist et al. 1983; Land & McCann, 1971; Marr, 1982), which sought to decompose the retinal image into components of perceived surface lightness and illumination that mirror the physical variables of reflectance and illumination that had combined to produce the retinal image in the first place. These are informally called layer theories.

Later, as the failure of the decomposition theories to explain lightness illusions (Agostini & Galmonte, 2002; Agostini & Proffitt, 1993; Bressan & Actis-Grosso, 2001; Economou et al. 2007; Soranzo & Agostini, 2006a,b) and failures of lightness constancy (Gilchrist, 1988) became clear, some theorists abandoned the inverse optics approach and proposed new, mid-level, so-called framework theories (Adelson, 2000; Gilchrist et al. 1999) that featured illumination frames of reference.

Meanwhile low-level approaches based on lateral inhibition became more sophisticated as they morphed into, for example, the neural/computational models proposed by Grossberg and Mingolla (1987), Grossberg and Todorovic (1988), and Pessoa, Mingolla, and Neumann (1995); or the spatial filtering models proposed by Blakeslee and McCourt (2001), Kingdom and Moulden (1992), and Watt and Morgan (1985). But these, strictly speaking, are models of brightness (perceived luminance) not lightness (perceived reflectance). The important problem is how we perceive the properties of objects (like reflectance), not how we perceive the properties of the light entering the eye.

Both layer and framework theories attempt to explain surface lightness, and both assume that the retinal image is parsed into components. But they propose different components. The debate between these layer and framework theories is the focus of the present paper.

## Decomposition theories

The theories of lightness that arose in the 1970s with the cognitive revolution generally sought to explain veridicality, not error, using an inverse optics approach to decompose the image into its reflectance and illuminance layers (Adelson, 1993; Adelson & Pentland, 1996; Arend, 1994; Barrow & Tenenbaum, 1978; Bergström, 1977; Gilchrist, 1977, 1979; Gilchrist, et al., 1983; Land & McCann, 1971; Marr, 1982). Unlike the earlier low-level explanations that took absolute luminance as the input, these theories generally assumed that only luminance ratios at edges are encoded at the retina. Gilchrist (1979; Gilchrist & Jacobsen, 1983; Gilchrist, et al., 1983) and Arend (1994) suggested that luminance ratios at edges are extracted from the retinal image and classified as changes in either reflectance or illumination. All the reflectance edges are then integrated to form a map of surface reflectance across the visual field and the illumination edges are integrated to form an additional map of the overlying pattern of illumination on the scene (Gilchrist, 1979; Gilchrist et al., 1983). Barrow and Tenenbaum (1978) called these layers intrinsic images.

**Fig. 1:**
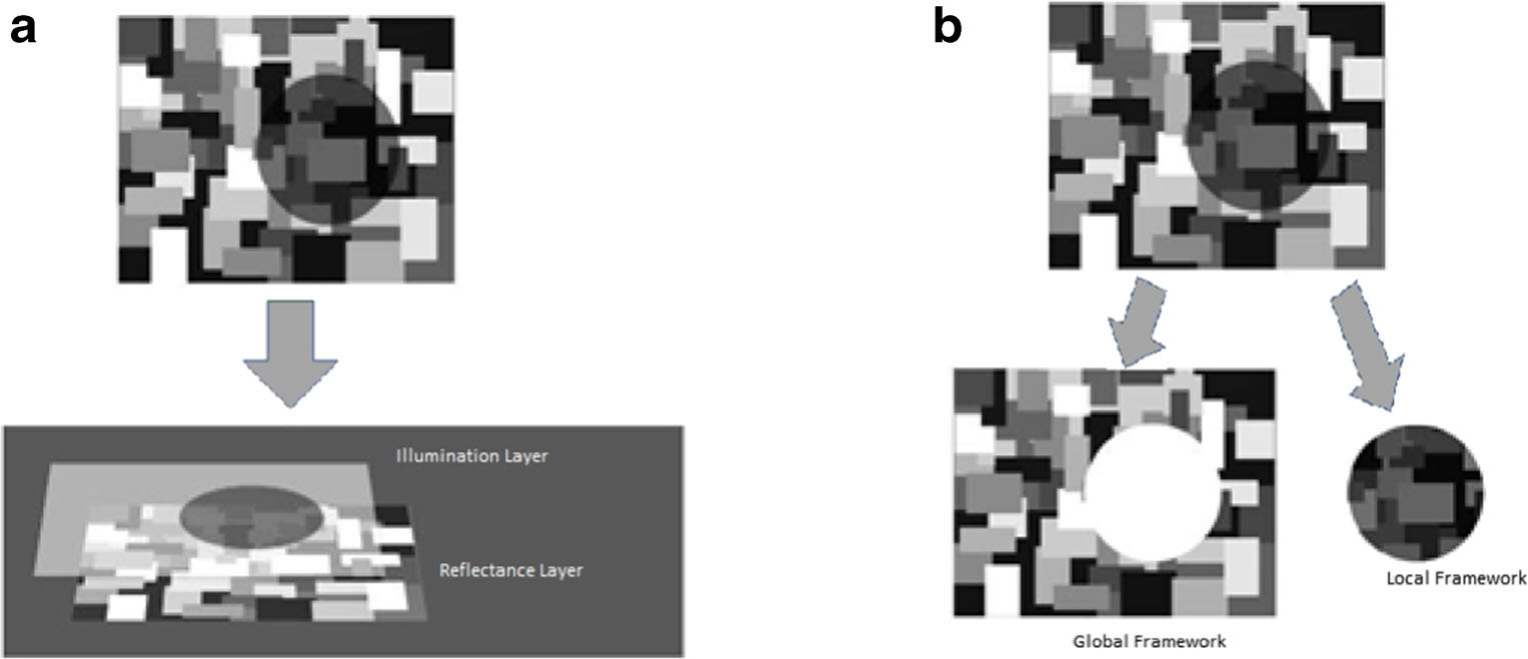
**(Left, a)**: Layer theories split the retinal image into a layer of perceived illumination projected onto a layer of surface reflectance. **(Right, b):** Framework theories parse the retinal image into frameworks of illumination bounded by corners, occlusion boundaries, and penumbrae

Figure 1 (left, a) represents how the visual system decomposes luminance in a bipartite field of illumination according to the decomposition approaches.

If the layers are parsed successfully, perception is completely veridical and no errors are predicted. But in that case the decomposition approach could not explain either failures of constancy or illusions, such as simultaneous lightness contrast (gray patch on a white background appears darker than identical patch on black background). Seeking to accommodate these so-called errors, several authors (Gilchrist, 1988; Ross & Pessoa, 2000; Soranzo & Agostini, 2004; Soranzo et al. 2009; Soranzo, Lugrin, & Wilson, 2013; Spehar et al., 2002) proposed the idea of partial classification of luminance edges. Lightness constancy errors are explained by assuming that the illumination edges are not fully excluded from the integration, but partially encoded as reflectance changes. Ironically, these attempts have been only partially successful.

### Other decomposition theories

Other theories have much in common with intrinsic image theories even if they don’t speak in terms of overlapping layers. Bergström (1977) proposed that by means of an analysis into common and relative components, the light composing the retinal image is decomposed into changes of reflectance, illumination, and 3D form. Musatti (1953) had earlier proposed a very similar idea using the now-confusing terms assimilation and contrast. Adelson and Pentland (1996) offered a vivid metaphor in which a workshop constructs a theatre set using workers who paint surfaces, configure lighting, and bend metal, mirroring Bergström’s three components. Brainard and Maloney (2011) have proposed an equivalent illumination model, suggesting that constancy fails when the illumination is misperceived.

Although strictly speaking these theories are not layer theories, they imply that the retinal image is experienced as a layer of illumination projected onto a layer of surface reflectance. More importantly, they share with the intrinsic image theories the crucial idea of complementarity between perceived illumination and perceived reflectance, whether or not there is an error in the attribution of the luminance to the different components. When a paleontologist cracks open a layer of rock to reveal a fossil, the two resulting layers of rock are exactly complementary to each other, even if the split does not fall exactly along the surface of the fossil. Likewise, when the visual system splits the luminance, the two resulting layers of lightness and perceived illumination are complementary to each other, even if the split does not correspond exactly to the actual proportions of reflectance and illumination. This implies that when a higher amount of luminance is attributed to lightness a complementary smaller amount of luminance is attributed to illumination. This idea of complementarity can be found in Koffka (1935; page 244) who suggested "the possibility that a combination of whiteness and [perceived illumination], possibly their product, is an invariant for a given local stimulation under a definite set of total conditions. If two equal proximal stimulations produce two surfaces of different whiteness, then these surfaces will also have different [perceived illuminations], the whiter one will be less, the blacker one more [brightly illuminated]." (For clarity we have substituted the term “perceived illumination” for Koffka’s term “brightness”, which meant perceived illumination then, but currently means perceived luminance.)

## Framework approach

Attempting to account for both lightness constancy and its failures, several theorists have moved away from the inverse optics logic of the decomposition approach toward theories that feature frames of reference but are also more rough and ready. Gilchrist abandoned his inverse optics approach and proposed a new anchoring theory (Gilchrist et al., 1999; Gilchrist, 2006). Commenting that “the Helmholtzian approach is overkill” Adelson (2000, p. 344) also shifted towards a frameworks approach. Similarly, Anderson abandoned Metelli's (1974) inverse optics approach in favor of a mid-level theory of transparency (see, e.g., Anderson & Khang, 2010).

### Anchoring theory

Helmholtz (1925) suggested that in order to compute lightness, the illumination level must be taken into account. However, according to the anchoring theory (Gilchrist, 2006; Gilchrist et al., 1999) the visual system doesn’t need to know the actual level of illumination – it only needs to know which surfaces are getting the same amount of illumination. This translates the problem into one of perceptual grouping. Multiple surfaces perceived as equally illuminated can be called illumination groups, and these are products of both grouping and segmentation processes. For example, in Gilchrist’s work on depth and lightness, these groups coincide with perceived planes. Co-planar surfaces can be said to be grouped by co-planarity or, alternatively, segregated by depth boundaries. Such groups of equi-illuminated surfaces function as frames of reference (Duncker, 1938; Koffka, 1935).

The lightness of a given surface is computed relative to its frame of reference, but not exclusively! Lightness is a weighted average of the lightness computed for a target strictly within its local framework (such as the region within a shadow) and its lightness computed in relation to the entire (global) visual field (Gilchrist et al., 1999). This scheme is roughly equivalent to an earlier proposal by Kardos (1934) called co-determination, in which lightness is seen partly in relation to its relevant framework and partly to the foreign framework. The weighting of local and global values depends primarily on the number of elements, or articulation level (Katz, 1935), of the local framework. Within each framework, lightness is anchored by assigning white to the highest luminance and evaluating every other shade relative to that, using the formula:

$$ Perceived\ reflectance= target\ luminance/ highest\ luminance\times 90\% $$

Bressan (2006) has proposed an alternative version of anchoring theory called double-anchoring theory, which includes a surround-as-white rule of anchoring.

Adelson’s (2000) newer approach is based on the concepts of atmospheres and adaptive windows. An atmosphere is a region of the visual field sharing the same illumination or glare or fog. Each adaptive window has its own atmosphere, and lightness estimates are computed based on the statistics within the window. The window is adaptive because its size changes as a function of the number of surfaces in a given area of the image. The window needs to be large enough so that it includes a sufficient number of samples. But if the window is too large it will include samples from different atmospheres. This theory is similar to the anchoring theory and it gives a great importance to the number samples in each adaptive window: the window grows when there are too few samples and shrinks when there are more than enough. In Gilchrist’s theory, a local framework coincides with illumination boundaries, while in Adelson’s theory the adaptive window has a soft boundary that doesn’t necessarily coincide with illumination boundaries.

## Comparison between the two theories

Layer and framework approaches parse the retinal image in different ways. While layer theories split the retinal into two overlapping layer-images, framework theories parse the image into adjacent regions of differing illumination level. At first glance, these two ways of decomposing the image appear incommensurate, even orthogonal. But each theory seems to offer important insights and this suggests that reconciliation might be possible.

Figure 1b represents how the visual system parses the scene into two fields of illumination according to anchoring theories. As one can see by comparing Fig. 1a and b, the components into which the image is decomposed are quite different for layer and framework theories. In anchoring theories, the image is segmented into frameworks using illumination boundaries (cast edges, occlusion edges, and corners) to parse the image into "contiguous regions of illumination or shadow, like states on a map" (Gilchrist, 2006; page 331), whereas in the intrinsic image approach, the image is split into complementary overlapping layers, one composed by integrating all the illumination edges, the other composed of all the reflectance edges.

Consider some crucial strengths and weaknesses of each approach.

**Fig. 2:**
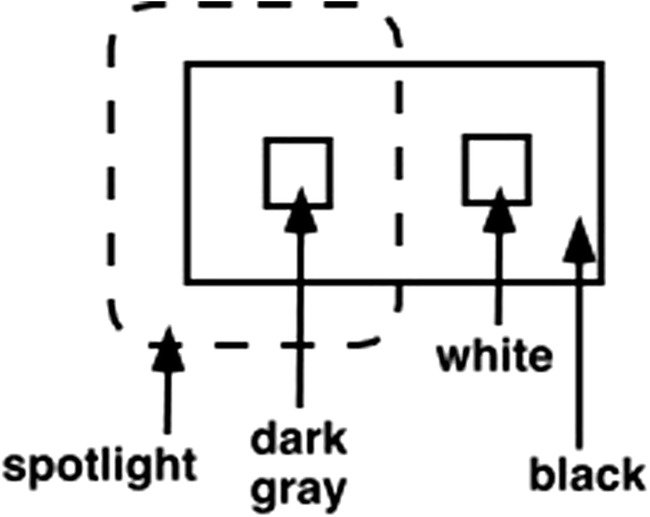
In this display (Gilchrist et al., 1999; p. 828) the dark gray target is the highest luminance in the display, and it appears lighter than the white target, which would be the highest value in the reflectance layer

1. *The anchoring problem*: Both layer and framework theories emphasize that lightness is associated with relative luminance. But the problem of how relative luminance values in the image are transformed into specific lightness values has been dealt with explicitly only by framework theories. According to empirical work the highest luminance is automatically anchored to white (Li & Gilchrist, 1999). In general, decomposition theories have not offered a satisfying solution to the anchoring problem. In support of a decomposition approach, Kingdom (2011) and Rudd (2013, 2014) proposed that the highest value in the reflectance layer, that is the highest lightness, is anchored to white. Rudd (2014) proposed an alternative anchoring rule, the highest reflectance anchoring specifying that the “highest reflectance in the scene always appears white, but the highest reflectance may not always be the same as the highest luminance” (p. 8). The author underlined that it is the largest reflectance in the neural representation, rather than the largest reflectance in the world, that appears white. However, Gilchrist et al. (1999) reported a test, shown in Fig. 2, pitting highest luminance against highest lightness, with results clearly supporting highest luminance. Due to the presence of the spotlight, the dark gray target on the left had the highest luminance in the display and it appeared fully white. The white target, although it had the highest value in the reflectance layer, appeared only light gray.

Zavagno et al. (2004) noted that the phenomenon of self-luminosity directly contradicts the claim that the highest luminance appears white. This apparent contradiction has been resolved by evidence that, in addition to relative luminance, lightness is anchored by relative area, with a tendency for the largest area to appear white (Bonato & Gilchrist 1999; Gilchrist 2006; Li & Gilchrist, 1999). Gilchrist (2006) introduced a distinction between upward and downward induction. When the luminance difference between a target surface and its surround increases, and the higher luminance occupies a much larger area than the lower luminance, the change is expressed as a darkening of the darker region, with little change in the lighter region. When the darker region occupies a much larger area, then an increase in luminance difference is expressed mainly as a trend towards self-luminosity.

2) *The scaling problem*. While the anchoring problem concerns which value of relative luminance is tied to which point on the lightness dimension, the scaling problem deals with how distances along the luminance scale are mapped onto distances along the lightness dimension. Decomposition theories have generally assumed that log luminance differences map directly onto log lightness differences (i.e., log-matched reflectance differences) in a 1:1 fashion. But lightness differences can be either compressed or expanded relative to luminance differences. Testing a high dynamic range Mondrian, Radonjic, et al. (2011) found a dramatic compression of the range; a 2,500:1 luminance range was perceived as spanning only a 30:1 reflectance range. Other work has shown a robust expansion; Ivory et al. (2011) found that a 5:1 luminance range Mondrian was perceived as spanning almost a 30:1 reflectance range. This is attributed by anchoring theory to scale normalization, a tendency to perceive the canonical range from white to black (30:1) within a framework. Expansion and compression of the gamut has not been addressed, and is not easily accommodated by decomposition theories.

**Fig. 3:**
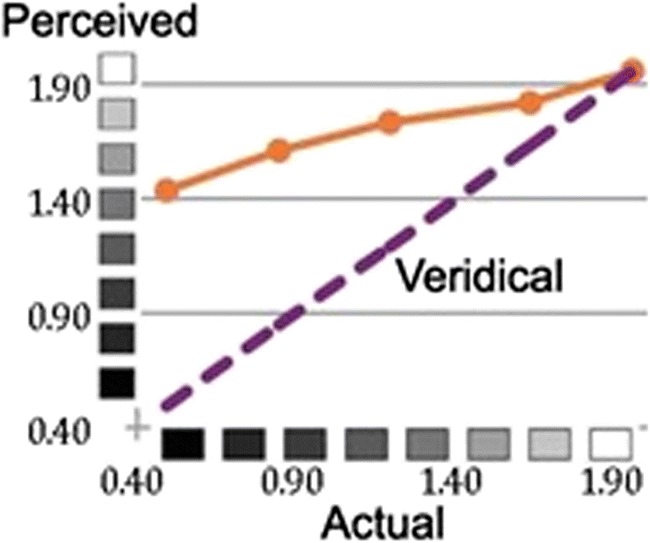
Staircase Gelb illusion. When a series of squares, ranging from black to white, is suspended in mid-air and illuminated by a spotlight, the perceived shades of gray are dramatically compressed relative to their actual shades

In 1995, Cataliotti and Gilchrist reported a dramatic new illusion dubbed the staircase Gelb effect. A row of five squares ranging from black to white was suspended in mid-air and illuminated by a bright spotlight. Not only did this arrangement produce a huge failure of constancy, with the black square appearing light gray, but more importantly, it introduced a strong compression of perceived reflectances relative to actual (see Fig. 3). In this case the compression cannot be attributed to scale normalization because it goes in the opposite direction.

Gamut compression in the staircase Gelb illusion poses a challenge to layer theories because the lightness of each square is shifted upward by a different amount. An error in decomposition – for example, if a portion of an illumination edge were erroneously classified as a reflectance change or if some portion of the common component were erroneously attributed to surface reflectance – would produce an offset in lightness values, rather than compression. In the spotlight, all reflectances should appear to be lightened by the same amount, but not compressed. Gamut compression is associated with the juxtaposition of two fields with differing levels of illumination, especially when one of the fields is smaller in area and/or is surrounded by the other. An analogous sort of gamut compression is found in a shadowed area, except that the compression is downward: high reflectances in shadow deviate from constancy more than low reflectances (Ivory & Gilchrist, 2010).

According to anchoring theory, the compression stems from what Kardos (1934) called co-determination; the lightness of a surface is not determined exclusively within its own framework of illumination but is partially anchored relative to the adjacent or surrounding field of illumination. Gilchrist and colleagues (Gilchrist et al., 1999) have argued that, although each of the five shades is computed veridically within the spotlight, they are all computed to be white relative to the surrounding dimmer illumination. The compression is held to result from the averaging of these equal lightness values together with their veridical values.

However, recent data from the Gilchrist lab has contradicted a prediction based on this account. When spotlight intensity is increased beyond 30 times the room level, no further compression should occur. With all shades computed as white relative to the global anchor, the source of compression is maxed out. However, experiments have shown (Gilchrist & Ivory, 2013) that the compression continues to increase as the spotlight intensity is increased beyond 30 times the room level, but that the degree of gamut compression is predicted by the luminance ratio between the highest luminance in the spotlight and the highest luminance in the surrounding field of illumination. This finding supports the general idea of co-determination while rejecting the particular mechanism proposed in the original version of anchoring theory (Gilchrist et al., 1999).

It has been argued (Allred & Brainard, 2013; Bloj et al., 2004) that gamut compression can be accommodated within the decomposition approach by assuming the perception of a different level of illumination on each patch within the shadow. But this construction defies the intuitive concept of illumination as dispersed across multiple surfaces.

3) *Role of articulation.* One of the most powerful factors in lightness is articulation, which refers to the number of distinct patches within a field of illumination. In their early work, Katz (1935) and Burzlaff (1931) found poor constancy when subjects matched a disk in bright illumination to a disk in an adjacent field in shadow. But they found almost perfect constancy when each field contained a tableau of 48 patches. Although this fact was forgotten during much of the prior century, many experiments in recent decades have confirmed it (Agostini & Galmonte, 1999; Arend & Goldstein, 1987; Cataliotti & Gilchrist, 1995; Schirillo & Arend, 1995). This is easily accommodated by the anchoring theory, with articulation level determining the weight of each competing framework in the co-determination process. But it is difficult to see how the role of articulation can be accommodated by a layer approach. In Gilchrist’s intrinsic image theory, for example, the degree of constancy found between adjacent fields of illumination should depend simply on how veridically the illumination edge between them is encoded and classified. The number of distinct patches within each field should play no role.
4) *Area effects.* Katz (1935) also found that the degree of constancy is influenced by the size of the field of illumination; hence his laws of field size. Such effects make sense within a framework approach because the area of a field influences the weight of that field in the co-determination process. But, just as with articulation effects, area effects do not seem commensurate with layer theories.

Layer theories are associated with the idea of inverse optics. So, for example, when a shadow falling across a scene introduces a luminance step into the image, that step is classified as a change of illumination, and excluded from the reflectance map. In other words, the confounding of illumination and reflectance in the formation of the image is inverted by edge classification. But effects of articulation and area have no counterpart in image formation. They do not function to invert or disentangle confounded variables. For example, if surfaces in the real world reflected more light as they become larger in area, then the effect of area on lightness would be consistent with a decomposition approach. But that is not the way the world works.

5) *Transparency and veiling luminance*. Obviously, perception of opaque surfaces seen through a transparent layer favors a layer decomposition approach because the scene contains spatially separated layers. Indeed, Metelli’s (1975) work on transparency was an early and explicit inverse optics approach, with perceptual color scission the inverse of color fusion. The transparent surfaces he studied include both a filter and a veil component. But a veiling luminance can occur without a filter component, as when looking through a sheet of light reflected in a window. Glare also constitutes a veiling luminance in the eye. When a veiling luminance is superimposed on a scene, both luminance values and luminance ratios are altered. Nevertheless, empirical results show an impressively high degree of lightness constancy through a veiling luminance (Gilchrist & Jacobsen, 1983). Both layer and framework theories work well for the filter component, like the shadow it mimics (Metelli, 1974, 1975), but the veil component is better handled by a layer theory.
6) *Black rooms and white rooms.* Gilchrist and Jacobsen (1983) obtained lightness matches for two miniature rooms filled with abstract 3D objects. One was painted entirely black, including the contents, and the other was painted entirely white. The white room was perceived as completely white, while the black room was perceived as a middle gray, a result that is more consistent with a layer theory than a framework theory. Because all of the luminance edges were either gradual or, when sharp, coincidental with a change of planarity, they were perceptually attributed to the illumination. Presumably the removal of these edges from the image leaves only a homogeneous reflectance layer. But the specific lightness value of that layer is not specified. The same challenge applies to shape-from-shading. Algorithms exist that transform the luminance variations across the image of a sculpture into a specific three-dimensional shape with homogeneous lightness (Horn, 1975, 1977; Pentland, 1989). But they do not specify that lightness value.

Perception in a three-dimensional world of one reflectance poses a challenge for a framework theory as there are no obvious frameworks. One could treat each plane as a framework but such a framework would have the minimum articulation level of one.

Gilchrist and Jacobsen (1983) showed that the luminance variations across the white room and the black room were qualitatively the same, but with much greater amplitude (i.e., high contrast) in the black room. Recent work by Gilchrist and Ivory (2011) further isolated this factor. They found that reducing the variation amplitude by covering a black room with a veiling luminance makes it appear as a white room.

But why does the black room (without the veil) appear middle gray rather than black? Could this appearance be the result of a compromise between the highest luminance rule of anchoring (indicating a white room) and the high-contrast luminance variations (indicating a black room)? This would be consistent with the spirit of compromise in the anchoring theory, but without the competing frameworks.

Adelson (2000, p. 346) has observed that X-junctions that emerge where the edge of a veiling luminance intersects a more distant reflectance edge creates the impression of a haze or veil. Although Anderson and his co-authors (Anderson, Singh, & Meng, 2006; Anderson & Winawer, 2005, 2008; Anderson, 1999, 2003) are leading proponents of layer theories, they have proposed a Transmittance Anchoring Principle (TAP), for discovering what part of a scene is covered by a veiling luminance. TAP states that the “visual system treats the highest contrast image regions as regions in plain view and only infers the presence of transparent surfaces if there are spatial or spatio-temporal perturbations in the contrast magnitude along contours, surfaces, or textures” (Anderson & Winawer, 2008; page 5).

### What are the possibilities for integrating layer and framework approaches?

Phenomenology offers important guidance but does not seem to resolve our dilemma. We do seem to perceive a layer of illumination projected onto a layer of surface reflectance. But we also experience frameworks of illumination. This suggests that an integration of the two kinds of theory is possible.

There is no contradiction between layers and frameworks, but rather between layer theories and framework theories. It can be said that layer and framework theories parse the retinal image into different components, i.e., layers and frameworks. But this is a bit misleading. For framework theories, the process begins with segregation of the image into frameworks. But for layer theories, the reflectance and illuminance layers represent the end of the process, not the beginning. Layer theories begin with edge encoding and edge classification (Gilchrist et al., 1983). Of course, edge classification and framework segregation are very closely related. If you know where the illumination edges are, you know where the frameworks are.

Both layer and framework approaches have a gestalt flavor. Both accept relative luminance as an input. Neither approach is structure-blind. Both find support in Koffka (1935; see also Gilchrist, 2006, pp. 371-373). The concept of frames of reference was central to Koffka’s thinking, which is most clearly revealed in his analysis of motion perception. But he also treated different regions of illumination as frameworks. Like Kardos (1934), Koffka did not consider the lightness value of a target to be exclusively computed within its own framework, but recognized that values within one framework are distorted by the presence of adjacent or surrounding frameworks. This can be seen in his remark that "a field part x is determined in its appearance by its 'appurtenance’ to other field parts. The more x belongs to the field part y, the more will its whiteness be determined by the gradient xy, and the less it belongs to the part z, the less will its whiteness depend on the gradient xz" (page 246).

This graded account of belongingness is consistent with the idea of co-determination (Kardos Gilchrist), whereas layer theories seem to require more of an all-or-none kind of belongingness in which a target belongs exclusively to one framework or another. At the same time, Koffka’s lightness/perceived illumination invariance theorem, according to which a combination of lightness and perceived illumination is invariant for a surface of constant luminance, seems equivalent to the complementarity implicit in the decomposition approach.

Empirical results have shown that both lightness (Li & Gilchrist, 1999) and perceived illumination (Gilchrist & Soranzo, under review; Kozaki 1965, 1973; Oyama, 1968) are anchored by highest luminance. Although this work was done primarily in the context of framework theories, these results imply the complementarity layer theories and Koffka’s theorem. Consider a group of patches, such as a Mondrian array. When a higher maximum luminance is added to the array, two things happen. First, the lightness of a given patch in the Mondrian goes down. Second, the perceived illumination on the Mondrian goes up. And as long as the Mondrian is well articulated, these upward and downward shifts are equal in magnitude. However, in the staircase Gelb illusion, with its relatively weak articulation, errors in lightness and perceived illumination are not equal and opposite. Gamut compression implies that the lightness error is different for each square in the staircase. Thus, any error in perceived illumination could be equal and opposite to, at most, the lightness error for a single square.

## Conclusions

From what we outlined above, it seems clear that both the decomposition and framework approaches to lightness perception have their pro and cons.

Layer theories do not have an adequate account of the anchoring problem. They cannot account for scaling effects, such as gamut expansion when viewing a low dynamic range Mondrian, gamut compression when viewing a high dynamic range Mondrian, or gamut compression in the staircase Gelb illusion. And they fail to capture lightness changes that occur due to an increase or decrease in the number of visible patches within a field of illumination. Framework theories have been quite successful in accounting for these effects.

On the other hand, framework theories cannot account for lightness constancy through a veiling luminance, although intuition suggests that this is a version of the scaling problem. Layer theories easily accommodate the experience of seeing a veil layer in front of an opaque surface layer, but they do not specify how the presence of the veil is detected and how the intensity of the veil is computed. Framework theories have so far said very little about perception of the illumination level, beyond the important finding that perceived illumination level is signaled by highest luminance (Kozaki 1965, 1973; Oyama, 1968). One concept central to both layer and framework theories is that of illumination edges. In layer theories, illumination edges are separated from reflectance edges while in framework theories, illumination edges form the boundaries of frameworks.

It seems that a new, more comprehensive theory is needed that is capable of integrating the two approaches. However, because the units into which the retinal image is split, layers versus frameworks, are so different, it is difficult to see how the two approaches can be integrated.
